# Improving Residency Matching Through Computational Optimization

**DOI:** 10.1001/jamanetworkopen.2025.17077

**Published:** 2025-06-23

**Authors:** Yue Wu, Cecilia S. Lee, Aaron Y. Lee, Russell N. Van Gelder

**Affiliations:** 1 Karalis-Johnson Retina Center, Department of Ophthalmology, University of Washington, Seattle; 2 Department of Laboratory Medicine & Pathology, University of Washington, Seattle; 3 Department of Neurobiology & Biophysics, University of Washington, Seattle

## Abstract

**Importance:**

In the US, tens of thousands of medical student applicants and residency programs rank each other annually. The matching algorithm, Gale-Shapley, has been relatively unchanged for over 50 years, and although it results in a stable solution for the match, where no applicant and program would prefer to be matched together than to their assigned match, it does not optimize the overall match result.

**Objective:**

To compare the performance of a mixed-integer linear optimization-based approach, the residency optimizer, for matching and against the currently used Gale-Shapley algorithm.

**Design, Setting, and Participants:**

This quality improvement study used anonymized rank lists and match data for ophthalmology residency matches conducted between 2011 to 2021. The Gale-Shapley algorithm and the residency optimizer were compared for overall performance for both applicants and programs, under both unlimited choice rank lists and capitated lists. The algorithms were also compared for couple matching. Final data analyses were performed in April 2025.

**Exposure:**

Matching with an ophthalmology residency and fellowship.

**Main Outcomes and Measures:**

Algorithm performance was compared in terms of mean matched rank of applicants and programs, as well as the percentage of applicants matching their top 3 ranked programs. For the capped rank list experiments, the percentage of positions filled and the percentage of applicants matching their top choices were measured. For couple matching, the percentage of couples matched was computed.

**Results:**

For a total of 6990 applicants (635.5 applicants per year) and a mean of 114.6 programs per year from 2011 to 2021, applicants matched 0.45 rank positions better (2.40 using the optimized algorithm vs 2.85 using Gale-Shapley), and the program matched applicants 0.32 rank positions better (2.65 for the optimized algorithm vs 2.97 for Gale-Shapley) per position under residency optimizer match than under Gale-Shapley. In total, 78.4% of applicants (4079 of 5200 applicants) matched to 1 of their top 3 choices with the residency optimizer match compared with 70.9% (3668 of 5174 applicants) with the Gale-Shapley algorithm. The residency optimizer eliminated unfilled residency positions and resulted in a mean of 2.4 fewer programs with unfilled slots annually. The residency optimizer outperformed the Gale-Shapley algorithm with truncated match lists and outperformed Gale-Shapley in successfully matching couples.

**Conclusions and Relevance:**

These findings suggest that alternate match algorithms optimizing global utility may generally improve residency and fellowship match outcomes.

## Key Points

**Question:**

Can the currently used Gale-Shapley residency match algorithm be improved to ensure more applicants and more programs match their top choices?

**Findings:**

Using a mixed-integer linear optimized algorithm with historical match list data from 2011 to 2021 for a total of 6990 applicants (635.5 applicants per year) and a mean of 114.6 programs per year, this quality improvement study found that the mean rank of programs for applicants was 2.40 using the residency optimizer, compared with 2.85 using Gale-Shapley; similarly, the mean rank of applicants for programs was 2.65 for the residency optimizer and 2.97 for Gale-Shapley. The residency optimizer consistently matched 5% to 10% more applicants to their top 3 ranked programs than Gale-Shapley.

**Meaning:**

These findings suggest that the Gale-Shapley algorithm produces a stable-marriage match, whereas the residency optimizer improves the match so that more applicants and programs match their top ranked counter-parties; thus, consideration should be given to updating residency match algorithms.

## Teaser

This quality improvement study compares the performance of a mixed integer linear optimization-based approach against the currently used algorithm for matching applicants to medical residency programs.

## Introduction

The National Resident Matching Program (NRMP) and other residency matching services such as the San Francisco Residency and Fellowship Match Services (SF Match) have used stable marriage algorithms^1^ to match students to residency programs without fundamental alteration since 1952. Gale and Shapley proved in 1962 that stable marriage algorithms are equivalent to their eponymous algorithm.^2^ The Gale-Shapley algorithm matches all participants from 2 groups into stable pairs, if there are an equal number of participants in both groups and if each participant ranks every potential partner. The matches are stable when no applicant and program would prefer to be matched together than to their assigned match.

Changing trends in the residency matching process over the years, however, challenge some of the idealized assumptions of the Gale-Shapley algorithm. For example, the numbers of applicants and residency positions are highly imbalanced.^3,4^ This has strained the match process over time, so that applicants apply to increasingly more programs, and programs likewise interview more applicants, increasing costs for all.^5^ The Nash equilibrium for the Gale-Shapley algorithm induces applicants to apply to, interview with, and rank as many programs as possible.^6^

The current match algorithms have other challenges. Although applicants outnumber residency positions, not all residency slots are filled^3^ because the applicants and the programs do not submit full rank lists of all counterparties. In 2020, there were 60 856 applicants for 34 266 postgraduate year one positions in NRMP, but only 32 399 positions were filled,^3^ leaving 1867 unfilled. These unfilled positions must then be filled through the Supplemental Offer and Acceptance Program for NRMP specialties.^7^ In addition, stable marriage algorithms require strict ordinal rank lists, which do not allow ties and fail to capture relative preferences. For example, an applicant may greatly prefer their top choice to their second choice program, but this preference is not captured in the rank list. It has been shown that ordinal ranks differ substantially from students’ marginal preferences.^8,9^ Gale-Shapley–style stable marriage algorithms also do not readily accommodate couples matching. NRMP requires couples to rank pairs of programs, which often prioritizes the career of one partner over the other. Although ranking 100 programs is rare for individual applicants, a couple in which each partner was interviewed by 13 programs (the median number of interviews for matched applicants) would need to rank 169 potential program pairs.

Since adoption of Gale-Shapley algorithms for the match over 60 years ago, there have been fundamental improvements in linear and nonlinear optimization computational methods. In this study, we describe a novel optimization-based method for matching applicants to residency programs, the residency optimizer (ResOpt), which maximizes the global utility of all participants without favoring either side. We compared this method with Gale-Shapley–based match results, using real-world retrospective data for the ophthalmology specialty residency match for the past decade.

## Methods

This quality improvement study did not need institutional review board approval because the data were fully deidentified for both applicants and programs. Informed consent was not needed because no individual participant data were used, in accordance with 45 CFR §46. This study follows the Strengthening the Reporting of Observational Studies in Epidemiology (STROBE) reporting guidelines.

### Study Datasets

Nine years (2011-2019) of anonymized rank lists for ophthalmology residency applicants and programs were provided by SF Match with permission of the Association of University Professors of Ophthalmology. Individual and program identities were not made available to the researchers. Additional out-of-sample datasets for 2020 to 2021 ophthalmology residency applicants and programs, as well as 2011 to 2020 ophthalmology fellowship applicants and programs, were provided by SF Match.

### Algorithms

The Gale-Shapley algorithm^2^ was implemented in Python version 3.5 (Python Software Foundation). For the residency optimizer, the residency match can be viewed as an output matrix of program slots and matched applicants resulting from 2 input matrices: (1) the applicants and their ranks of programs, and (2) the programs and their ranks of applicants. Utility is defined as the total sum of matched ranks for both applicants and programs (with the latter normalized to number of positions per program). The residency optimizer was designed to maximize the global preferences for both students and program by identifying the minimum in the utility function.

### Statistical Analysis

Final data analyses were performed in April 2025. The residency optimizer was implemented using mixed-integer linear programming. The mixed-integer linear programming solver takes the following inputs: (1) a *p* × *n* matrix *f*, that represents the sum of the applicant and program rank preferences, and (2) constraints. For the residency matching problem, *p* and *n* are the number of programs and applicants, respectively. The solver determines the *p *× *n* match matrix *x* that minimizes the sum of element-wise multiplied *f* and *x*, subject to the constraints. Element *x_ij_* is 1 if applicant *j* matched to program *i*, and 0 otherwise. The constraints are that (1) no applicant matches more than 1 program, and (2) no program matches more their number of available spots.

To compare the residency optimizer directly with Gale-Shapley, *f* was chosen to be the sum of the applicant ranks and normalized program ranks. Normalized program ranks are the raw program ranks divided by the number of program spots, which makes programs of different sizes more equitable. For example, an applicant ranking a program second and a 5-spot program ranking an applicant 10th should have the same amount of impact in the optimizer, since in effect the program ranked the applicant among its second tier of candidates. Similarly, a 6-spot program ranking an applicant 18th and a 3-spot program ranking an applicant ninth will have the same normalized rank of 3.

#### Truncation Analyses

The robustness of residency optimizer and Gale-Shapley to shorter rank lists, and thereby fewer interviews for applicants and programs, was analyzed by truncating the rank lists of programs and applicants from 2011 to 2019. Rank lists were truncated from 0% to 95%, in incremental steps of 5%. For example, a truncation level of 10% for programs would remove the bottom 10% of ranks from each program rank list, while applicant rank lists were not truncated. Similarly applicant lists were truncated while leaving program lists unchanged. Finally, both applicant and program rank lists were truncated. The Gale-Shapley and residency optimizers were then applied to the truncated rank lists. The fraction of positions filled was computed for each truncation level for both algorithms.

#### Fellowship Matching

The residency optimizer was developed on the 2011 to 2019 ophthalmology residency match data. It was applied to the 10 years (2011-2020) of ophthalmology fellowship match data, without any modifications to the algorithm.

#### Couples Matching

The residency optimizer was adapted for couples matching in the same city, by adding a constraint for each couple, denoted (*i*, *j*), to the optimizer. The constraint is that the difference in columns *x_i_* and *x_j_* for programs in the same cities is 0, so that either both partners matched in the same city or did not match. The residency optimizer requires no paired ranking of programs as must be done presently in NRMP, but may be customized for each couple, by specifying which programs they consider adjacent to avoid situations where programs are nominally in the same city but have long commutes.

To compare residency optimizer with city matching (ResOptCity) vs Gale-Shapley, 1, 8, and 16 couples were created by randomly sampling 1 partner from the 2019 ophthalmology rank lists, and creating the other partner by mirroring the sampled partner’s program ranks. The programs then randomly ranked the created partner. ResOptCity and Gale-Shapley were run on the created rank lists. The experiment was repeated 1000 times for different numbers of couples.

#### Residency Optimizer vs Gale-Shapley in NRMP Specialties

To verify that our results were not unique to the ophthalmology match, we varied numbers of applicants and program slots to simulate 10 NRMP specialties of different competitiveness: dermatology, family medicine, general surgery, internal medicine, orthopedics, otolaryngology, pathology, plastics surgery, and radiology. As NRMP does not make its match datasets available, we imputed rank lists for these specialties. Using the 2019 ophthalmology rank lists as starting points, the NRMP rank lists were bootstrapped to match the 2019 published mean statistics,^10,11^ including number of programs, number of positions, number of applicants, and the mean number of programs ranked per applicant (eTable 1 in Supplement 1). In addition, other rank list characteristics, such as distribution of rank list sparsity, distribution of program popularity, distribution of program desirability, distribution of applicant popularity and desirability, were modeled and retained. Distributions of popularity and desirability were modeled separately, as programs or applicants might be popular and be ranked by many counterparties, but not necessarily desirable if they were not ranked highly by counterparties. Details of the bootstrap process are provided in eAppendix 1 in Supplement 1. Gale-Shapley and residency optimizer were applied to these bootstrapped rank lists, and their resulting matches compared. Bootstrap experiments were repeated 10 times for each specialty.

#### Residency Optimizer vs Gale-Shapley for Specialty With More Non-US Senior Applicants

Another difference is that some NRMP specialties have a much larger percentage of non-US senior applicants compared with ophthalmology. For example, 60% of applicants in internal medicine are non-US seniors.^10^ To understand this effect, we randomly sampled 60% applicants from the 2019 ophthalmology rank lists, and reduced the number of programs ranking them by 30%, as the median number of ranks received by matching US seniors and non-US seniors in NRMP were 13 and 9, respectively, and 9 / 13 = 70%. Then the applicant and program rank lists were updated to be contiguous, and Gale-Shapley and residency optimizer were run. This experiment was repeated 10 times, and Gale-Shapley and residency optimizer were compared for fill rates, mean matched rank, and percentage of applicants matching their top 3 choice programs. Statistical significance was defined as 2-sided *P* < .05 by the paired Wilcoxon signed rank test. Data were analyzed with R statistical software version 4.0.2 (R Project for Statistical Computing). Additional methods are shown in eAppendixes 2 to 4 in Supplement 1.

## Results

The ophthalmology applicant and program rank lists for years 2011 to 2021 are summarized in the Table. On average, 635.5 applicants applied to 472.7 positions at 114.6 programs and filled 99.4% of these positions. The average applicant ranked 8.7 programs, and the average program ranked 11.7 applicants per position.

**Table.** Summary Statistics for Ophthalmology Rank Lists and Match Data

Year	No. of applicants	No. of programs	No. of spots	Applicants matched, No. (%)	Applicant ranked, mean (SD)	Program ranked per spot, mean (SD)
2011	634	114	461	457 (99.10)	8.23 (5.39)	11.03 (4.57)
2012	609	113	461	458 (99.30)	8.63 (5.14)	11.25 (4.50)
2013	591	113	460	455 (98.90)	8.78 (5.27)	11.10 (4.59)
2014	622	113	461	460 (99.80)	8.87 (6.51)	11.71 (5.18)
2015	654	113	465	464 (99.80)	8.89 (7.46)	11.66 (4.65)
2016	650	113	469	467 (99.60)	9.08 (6.54)	11.96 (4.88)
2017	600	112	468	462 (98.70)	8.99 (5.38)	11.64 (4.43)
2018	632	114	475	474 (99.80)	9.22 (6.80)	11.60 (4.23)
2019	665	116	485	484 (99.80)	8.53 (5.72)	11.54 (4.28)
2020	644	120	496	495 (99.80)	9.16 (5.32)	11.64 (4.67)
2021	689	120	499	498 (99.80)	9.21 (5.75)	12.41 (5.05)
Mean	635.5	114.6	472.7	470.4 (99.40)	8.66 (6.00)	11.69 (4.77)

### Utility of Residency Optimizer vs Gale-Shapley

**Figure 1.**
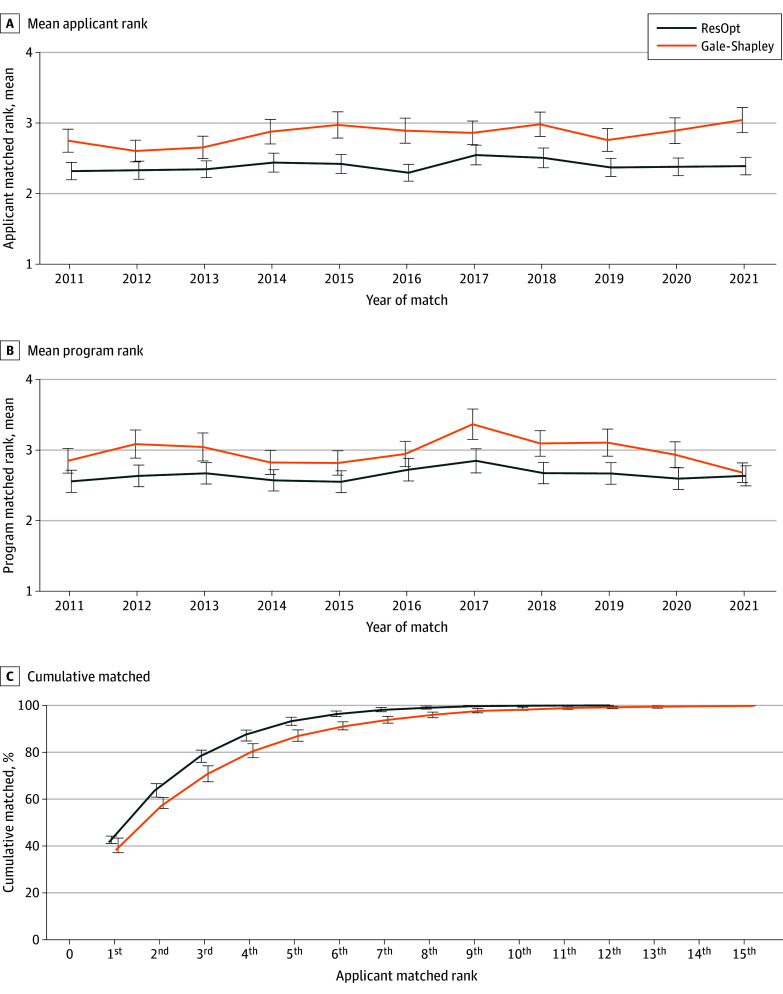
Match Outcome Using Gale-Shapley vs Residency Optimization Algorithm (ResOpt) Graphs show mean applicant rank of applicants’ matched programs (ie, 2.40 = mean rank position of matched program) (A), mean program rank of the programs’ matched applicants (ie, 2.65 = mean position on rank list per position of matched candidates) (B), and cumulative percentage of applicants matching into their top n^th^ program under ResOpt and Gale-Shapley (C). Error bars denote 95% CIs.

The residency optimizer ran in under 1 second on a standard laptop computer. The mean matched ranks resulting from residency optimizer were lower (ie, more desirable) for both applicants and programs across all years (Figure 1A and 1B). Tabular results are provided in eTable 2 in Supplement 1. For applicants, the improvement in mean ranks of matched programs is significant in most years as the 95% CIs do not overlap. Applicants matched 0.45 rank positions better (2.40 using the optimized algorithm vs 2.85 using Gale-Shapley), and the program matched applicants 0.32 rank positions better (2.65 for the optimized algorithm vs 2.97 for Gale-Shapley) per position under residency optimizer match than under Gale-Shapley.

For programs, the 95% CIs do not overlap in 4 of the 9 studied years. Over all 9 years, programs’ mean matched ranks under residency optimizer are significantly lower than under Gale-Shapley.

Residency optimizer matched more applicants to their top 3 program choices in all years than Gale-Shapley (Figure 1C and eTable 3 in Supplement 1). Over the 11 years, residency optimizer matched 78.4% of applicants (4079 of 5200 applicants) to their top 3 choices, vs 70.9% of applicants (3668 of 5174 applicants) for Gale-Shapley. The nonparametric 1-sided Wilcoxon rank sum test that the applicants’ matched program ranks is greater in Gale-Shapley than in residency optimizer. The parametric 1-way analysis of variance of applicants’ matched program ranks with residency optimizer vs Gale-Shapley was statistically significant. Both tests show residency optimizer significantly improves outcomes for applicants. Similarly, for the program side, residency optimizer matched significantly more programs to their more desirable candidates in the first 3 spots than Gale-Shapley (eFigure 1 in Supplement 1). The 1-sided Wilcoxon rank sum test that the programs’ matched applicant ranks is greater in Gale-Shapley than in residency optimizer and the 1-way analysis of variance of programs’ matched applicants ranks vs method both were statistically significant.

**Figure 2.**
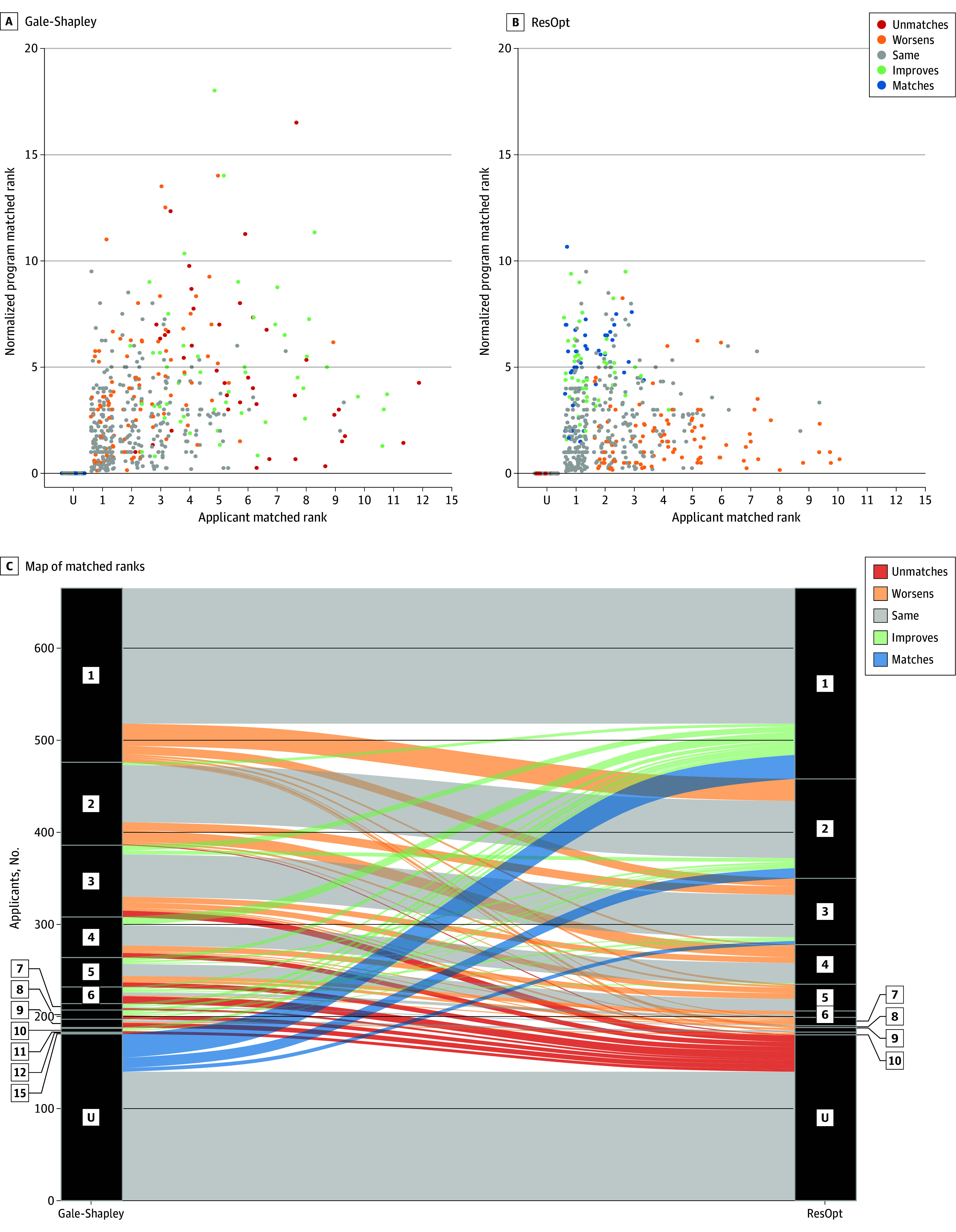
Matched Rank Comparison Between Residency Optimization (ResOpt) and Gale-Shapley Algorithms A and B, applicant matched ranks vs normalized program matched ranks with jitter in 2019 colored by their change in status between Gale-Shapley and ResOpt. On the x-axis, 1 represents applicants getting their first choice and U indicates unmatched. The y-axis shows rank per offered position. C, applicants’ matched ranks between Gale-Shapley and ResOpt in 2019 are mapped by rotating the x-axis in panels A and B and linking each applicant. Black numbers represent position on applicant match list.

Figure 2 illustrates the philosophical difference between Gale-Shapley and residency optimizer. Residency optimizer finds the optimal matches that minimize the sum of applicant and program ranks. Graphically, this means residency optimizer minimizes the spread of matches, squeezing them into a small triangular area (Figure 2B) compared with Gale-Shapley (Figure 2A). A majority of applicants in 2019 (296 of 485 applicants [61%]) matched exactly the same using either method (Figure 2C). However, when applicants matched more favorably, they did much better, going from less preferred programs to more preferred programs or matching under residency optimizer after being previously unmatched (Figure 2C). Applicants who did worse usually lost 1 rank, although some who had previously matched became unmatched. On average, an applicant who improved gained 4.1 ranks, vs 2.2 ranks lost for those who obtained a lower match than under Gale-Shapley. In total, 5.4% of applicants (377 of 6990 applicants) previously matched under Gale-Shapley became unmatched under residency optimizer, while 5.8% of previously unmatched applicants (403 of 6990 applicants) became matched. In addition, residency optimizer filled all open positions each year and resulted in a mean of 2.4 fewer programs with unfilled slots annually, while over the 11-year period, 26 residency slots went unfilled under Gale-Shapley.

### Truncation of Match Lists for Residency Optimizer vs Gale-Shapley

**Figure 3.**
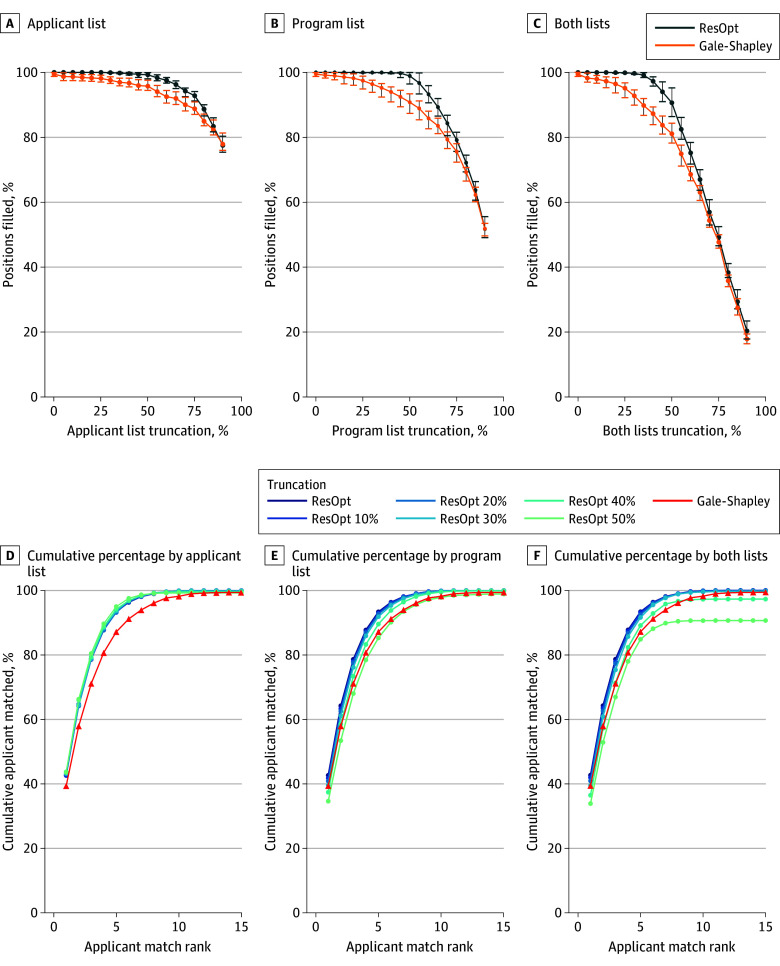
Percentage of Positions Matched at Different Truncation Levels for Gale-Shapley and Residency Optimization (ResOpt) Algorithms Graphs show percentage of positions matched for different truncation percentages of applicant rank lists (A), program rank lists (B), and both rank lists (C). The corresponding cumulative percentage of applicants who matched their top programs for these truncations are shown in panels D, E, and F, respectively.

As noted previously, the Gale-Shapley match filled 99.4% of positions (4181 of 4205 positions between 2011 and 2019), while residency optimizer filled all 4205 positions. Simulation of results from truncated match lists demonstrated that residency optimizer is more robust to shorter rank lists than Gale-Shapley (Figure 3). At 50% truncation of applicant, program, and both rank lists, residency optimizer matched 99.3%, 98.9%, and 90.7% of all available positions, respectively, compared with 95.8%, 90.8%, and 81.1% for Gale-Shapley. Residency optimizer performed with a 50% applicant or program list truncation at nearly the same overall match rate as Gale-Shapley performed with untruncated lists

Figure 3 shows the impact of truncation on the percentage of applicants matching their top choice programs. Applicant rank list truncation of even 50% had little effect on applicants matching their top choice programs under residency optimizer (Figure 3D), and still outperformed the baseline of Gale-Shapley with no truncation. Program rank list truncation had more impact, but truncation levels less than 50% still favored residency optimizer for the number of positions matched (Figure 3E). Truncation of both rank lists had a bigger effect on match outcomes, but with 30% or less truncation of both applicant and program lists residency optimizer still resulted in more matched positions than Gale-Shapley algorithm run on untruncated lists (Figure 3F). This result suggests that, under residency optimizer, both applicant and program rank lists may be shortened without adversely affecting overall match outcome, which could reduce applicant expense and program work to achieve largely similar results.

### Fellowship Matching

Unlike ophthalmology residency, which has low vacancy rates, more ophthalmology fellowships have higher unmatched rates (on the order of 10%-15%). Residency optimizer consistently filled 5% to 10% more ophthalmology fellowship positions than Gale-Shapley (eFigure 2 in Supplement 1) with similar overall utility. Residency optimizer outperformed Gale-Shapley particularly for less popular ophthalmology subspecialties (eFigure 3 in Supplement 1).

### Couple Matching

Couples matching with residency optimizer always matched successful couples in the same city, without hurting their overall chances of matching (eFigure 4 in Supplement 1). In contrast, Gale-Shapley only matched couples in the same city 40% of the time.

### Residency Optimizer for NRMP Specialties

**Figure 4.**
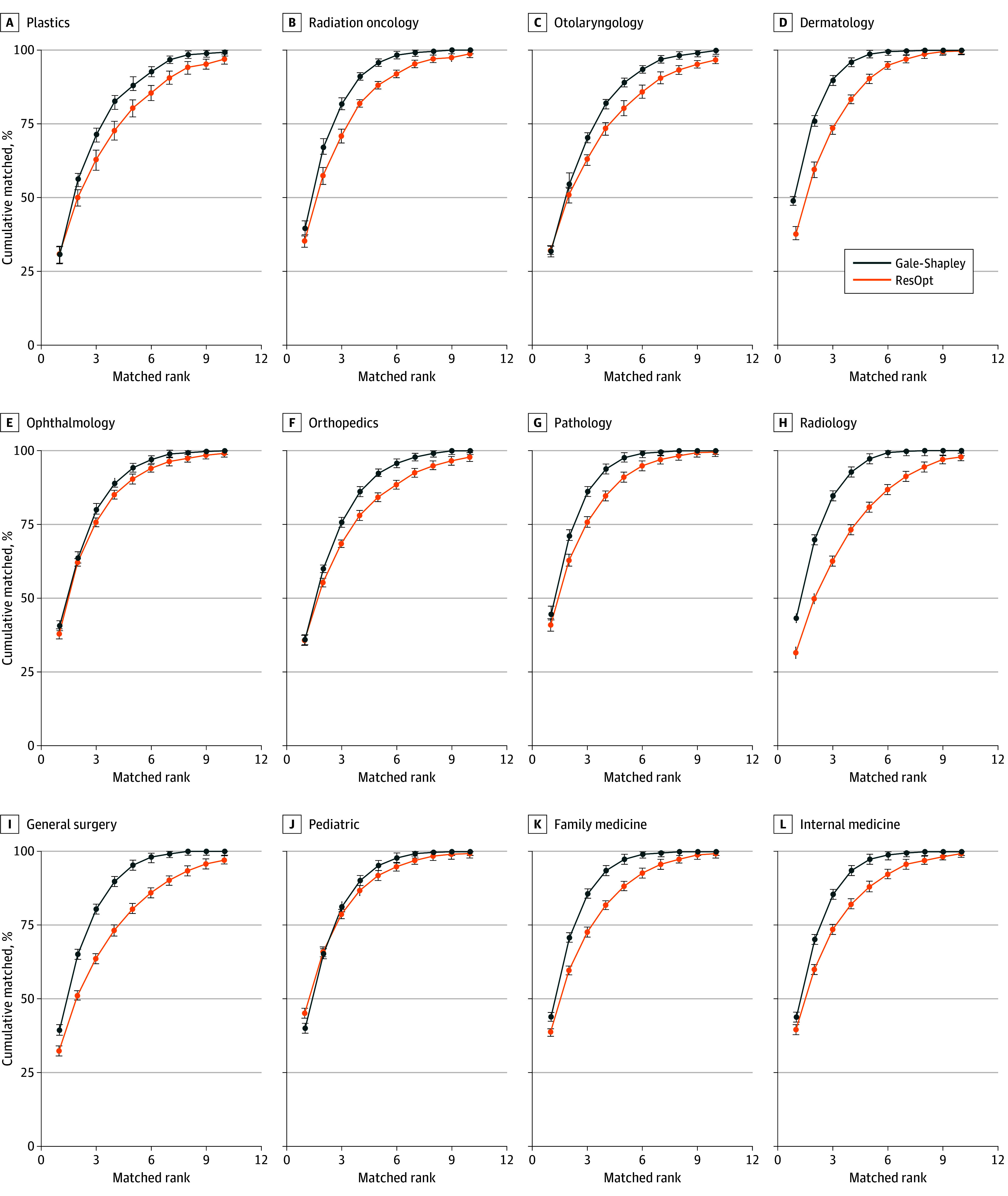
Cumulative Percentage of Applicants Matching Their Top n^th^ Choice by Specialty, for Gale-Shapley and Residency Optimization (ResOpt) Algorithms Averaged Over 10 Runs

Residency optimizer remained computationally efficient when run on larger matches, taking less than 10 seconds on a desktop computer to run for a simulated full NRMP match (see eAppendix 5 in Supplement 1). When simulated for other medical specialties, residency optimizer filled more positions than Gale-Shapley (eTable 4 in Supplement 1), filling 99.39% of positions on average vs 97.99% for Gale-Shapley. Moreover, residency optimizer achieved significantly better mean ranks of matched programs for applicants in all studied NRMP specialties (eFigure 5A in Supplement 1). In addition, residency optimizer achieved superior ranks of matched applicants for programs in most specialties (eFigure 5B in Supplement 1); for those specialties where programs had lower match ranking results, applicants fared much better. Finally, a higher percentage of applicants matched to their top choices under residency optimizer in all specialties (Figure 4). See additional data in eTables 5 to 9 and eFigures 6 and 7 in Supplement 1.

### Residency Optimizer for Non-US Seniors

When simulated for a larger non-US senior applicant pool, residency optimizer outperformed Gale-Shapley in terms of fill rate, lower mean matched ranks for applicants and programs, and more applicants matched to their top 3 choice programs than Gale-Shapley on all 10 in silico experiments (eTable 10 in Supplement 1). These results show that residency optimizer is more robust to scenarios where a certain group of applicants are less ranked than others.

## Discussion

The Gale-Shapley algorithm, which is the basis of the medical specialty match, is designed to produce a stable solution, wherein no applicant and program would prefer to be matched together than to their assigned match. Although this is one fundamental approach to the match, different match solutions could produce superior overall match results. In this quality improvement study, we have designed a mixed-integer linear optimization algorithm that finds the optimal solution to the matrix of applicants and programs based on their rank lists, with optimum defined as minimizing overall match rank (ie, what solution gives the overall lowest sum of applicant and program rankings). Using historical match rank lists, we found that this method resulted in improved overall match performance for both applicants and programs. Under residency optimizer, more applicants matched, more matched to their top choice programs, and more programs matched their top applicants. Furthermore, residency optimizer was more robust than Gale-Shapley, as it retained these characteristics even if the rank lists are shorter (as might occur in a situation in which applicant or program lists are limited or truncated).

The residency optimizer achieved improved outcomes by trading individual stability for global utility. Gale-Shapley iteratively considers one proposing applicant at a time, matching each to her or his most preferred program that has available spots. This is locally stable, but globally suboptimal. We have tested a constrained form of residency optimizer in which the match pool consists only of individuals who matched under Gale Shapley. Even in this situation, both applicants and programs attained superior matches under global optimization (data not shown). In toy problem small simulated matches (eAppendix 6 in Supplement 1), we found that overall match rank can be improved by more than 5% using rank optimization compared with Gale-Shapley methods.

Mixed-integer linear model optimization also outperformed Gale-Shapley when rank lists are shorter. The residency optimizer matched 99% positions even when rank lists were half as long, which was not the case for Gale-Shapley. Gale-Shapley induced applicants to apply to more programs, since by Nash equilibrium, applicants cannot do worse by applying, interviewing, and ranking more programs. Additionally, program rank lists are getting longer, as programs are risk averse to not filling all their spots under Gale-Shapley.^6^ This Nash equilibrium game theory strategic consequences are increased residency matching costs for both applicants and programs. In contrast to Gale-Shapley, residency optimizer does not induce these pressures, and can potentially make the residency matching process less expensive and more equitable for both programs and applicants by ensuring comparable performance under conditions of limited application and interview.

Unlike Gale-Shapley, residency optimizer can naturally incorporate couples matching by allowing couples to rank independently and only specifying which programs that they consider colocated. Couples do not have to be laboriously recoded to create strategically ranked ordered pairs of programs as is currently done in NRMP. Residency optimizer with couples matching achieves comparable match rates for couples as they would have achieved if matching individually.

It is likely that improvements on the order of one position in rank may be more beneficial to the applicant than to the program, as the desirability of a program to the applicant may be based on more than the academic quality of the program and may include considerations such as physical location, proximity to family, and so forth. For instance, an improvement from an applicant’s second-ranked program to first may have more value to the applicant than improvement from number 22 to number 21 on a program’s rank list. For the purposes of the current study, we have weighted these equally but this could be varied to favor either applicant or program more strongly. The results of the current study, however, suggest that at equal weighting of utility, improved applicant match under residency optimizer does not come at the expense of worse program match. Furthermore, other changes resulting from use of residency optimizer may be of greater utility to programs than applicants, such as the lower number of unfilled positions at the conclusion of the match.

### Limitations and Strengths

There are 3 primary limitations of the present study. First, the couples match results are based on synthetic data, and should be verified using real couples match data. Second, the NRMP and NRMP non-US senior results are based on bootstrapped data, and should be verified on real NRMP rank lists if possible. Furthermore, the impact of residency optimizer and Gale-Shapley should be analyzed vis-a-vis applicant ethnicity or race. eTables 6 and 7 in Supplement 1 show that percentages of applicants from minoritized racial and ethnic groups and acceptance rates were not dissimilar in ophthalmology and NRMP. Unfortunately, neither NRMP nor SF Match provides rank statistics demographically, to allow in silico comparisons. In addition, residency optimizer was run using the ordinal ranks as the utility to be more comparable to Gale-Shapley. This does not take full advantage of residency optimizer, which can incorporate relative preferences. However, relative preferences data would need to be collected to perform this analysis. We note that theoretically there could exist multiple optimal lowest rank configurations, in which case residency optimizer will only find one of those configurations depending on initial settings, such as random order in which rank lists are chosen. Similarly, Gale-Shapley will find only one stable solution depending on its initialization even if there are multiple stable solutions. In practice with real ophthalmology rank lists, there do not appear to be multiple optimal or stable marriage configurations, which was tested by varying starting conditions of residency optimizer and Gale-Shapley on the same dataset.

The residency optimizer system appears applicable to other specialty matching including larger and less competitive specialties. Despite potential differences in demographic and candidate characteristics such as non-US medical graduate status that may exist between ophthalmology and other specialties, our bootstrapping approach allowed us to replicate the summary match statistics for the model specialties. Additionally, we tried to model program and applicant desirability to assimilate the fact that competitive applicants are usually ranked at the top of most of the competitive program lists, causing a mismatch in any specialties. In each of these bootstrapped rank list simulations, residency optimizer resulted in better overall performance for both applicants and programs, and resulted in more applicants matching to one of their top choice programs. Nonetheless, it would be desirable to repeat our comparison with actual historical match data from all specialties to determine whether there are matches for which residency optimizer produces anomalous results or underperform relative to Gale-Shapely.

## Conclusions

The matching algorithms used by all graduate medical programs in the US have become entrenched in our nation’s process for assignment of specialty training. These algorithms, based on Gale-Shapley marriage problem solutions, have served well for over 50 years. However, advances in computational optimization now allow consideration of algorithms that can address deficiencies in Gale-Shapley, including suboptimal match assignment, excessive open match positions, challenges with couples matching, inability to incorporate nonordinal rank preference, and instability with shortened match lists. Consideration should be given to migrating match algorithms to global utility optimization-based methods.

## Notes

Supplement 1. **eTable 1.** Demographics for ophthalmology and selected NRMP specialties **eTable 2.** Match results for ResOpt vs Gale-Shapley **eTable 3.** Cumulative percentage of applicants matching their top three ranked programs by year for Gale-Shapley, ResOpt Constrained and ResOpt **eTable 4.** Average percentage of positions filled and standard errors under Gale-Shapley vs ResOpt for ophthalmology and selected NRMP specialties **eFigure 1.** The cumulative percentage of matches versus the program rank of the applicant under Gale-Shapley and ResOpt from the top matched to the sixth matched candidate if the program had six spots **eFigure 2.** Percentage of total ophthalmology fellowship positions filled by Gale-Shapley and ResOpt from 2011 to 2020 **eFigure 3.** Percentage of ophthalmology sub-specialty fellowship positions filled by Gale-Shapley and ResOpt from 2011 to 2020 **eFigure 4.** The percentage of times partner A, B matched individually, in the same program and in the same city under Gale-Shapley (GS) versus ResOptCity for 1, 8, and 16 couples or 0.3%, 2.5%, and 5% of the applicant pool respectively **eFigure 5.** The difference between applicants’ mean rank of their matched programs under Gale-Shapley and ResOpt **eAppendix 1.** NRMP bootstrap **eTable 5.** 2019 Reported total positions, matched positions, fill rate, versus fill rate and standard error for Gale-Shapley and ResOpt run on 10 bootstrapped rank lists **eAppendix 2.** Comparison of specialty demographics **eTable 6.** Applicant demographics for ophthalmology in 2019 and 2022 and available NRMP specialties in 2022 **eTable 7.** Applicant match rates for ophthalmology in 2019 and 2022 and available NRMP specialties in 2022 **eAppendix 3.** Comparison of educational background **eTable 8.** Number of matched applicants and their percentage of total matched applicants by schooling **eTable 9.** Number of applicants, percentage of all applicants, the number matches and the match rate for US graduating seniors versus all other sources for select specialties in 2019 **eAppendix 4.** Bootstrap of non-US senior applicants **eTable 10.** Fill rate, average matched ranks for applicants and programs as well as the percentage of applicants matching their top 3 programs for Gale-Shapley and ResOpt for 10 runs **eAppendix 5.** ResOpt run times **eFigure 6.** Number of programs times number of applicants versus CPU run times of ResOpt with error bars **eAppendix 6.** Toy problem **eFigure 7.** Rank lists for applicants A to F on the left and programs X to Z on the right
eReferences

Supplement 2.
Data Sharing Statement
