# Supplementary material for: Improving Residency Matching Through Computational Optimization
Source: JAMA Netw Open. 2025 Jun 23;8(6):e2517077. doi: 10.1001/jamanetworkopen.2025.17077 (PMC12186513; doi:10.1001/jamanetworkopen.2025.17077)
Supplement: Supplement 1. — eTable 1. Demographics for ophthalmology and selected NRMP specialties eTable 2. Match results for ResOpt vs Gale-Shapley eTable 3. Cumulative percentage of applicants matching their top three ranked programs by year for Gale-Shapley, ResOpt Constrained and ResOpt eTable 4. Average percentage of positions filled and standard errors under Gale-Shapley vs ResOpt for ophthalmology and selected NRMP specialties eFigure 1. The cumulative percentage of matches versus the program rank of the applicant under Gale-Shapley and ResOpt from the top matched to the sixth matched candidate if the program had six spots eFigure 2. Percentage of total ophthalmology fellowship positions filled by Gale-Shapley and ResOpt from 2011 to 2020 eFigure 3. Percentage of ophthalmology sub-specialty fellowship positions filled by Gale-Shapley and ResOpt from 2011 to 2020 eFigure 4. The percentage of times partner A, B matched individually, in the same program and in the same city under Gale-Shapley (GS) versus ResOptCity for 1, 8, and 16 couples or 0.3%, 2.5%, and 5% of the applicant pool respectively eFigure 5. The difference between applicants’ mean rank of their matched programs under Gale-Shapley and ResOpt eAppendix 1. NRMP bootstrap eTable 5. 2019 Reported total positions, matched positions, fill rate, versus fill rate and standard error for Gale-Shapley and ResOpt run on 10 bootstrapped rank lists eAppendix 2. Comparison of specialty demographics eTable 6. Applicant demographics for ophthalmology in 2019 and 2022 and available NRMP specialties in 2022 eTable 7. Applicant match rates for ophthalmology in 2019 and 2022 and available NRMP specialties in 2022 eAppendix 3. Comparison of educational background eTable 8. Number of matched applicants and their percentage of total matched applicants by schooling eTable 9. Number of applicants, percentage of all applicants, the number matches and the match rate for US graduating seniors versus all other sources for select specialties in 2019 eAppe [file jamanetwopen-e2517077-s001.pdf]

Supplemental Online Content

Wu Y, Lee C, Lee A, Van Gelder RN. Improving residency matching through computational optimization. *JAMA Netw Open*. 2025;8(6):e2517077.

doi:10.1001/jamanetworkopen.2025.17077

eTable 1. Demographics for ophthalmology and selected NRMP specialties

eTable 2. Match results for ResOpt vs Gale-Shapley

eTable 3. Cumulative percentage of applicants matching their top three ranked programs by year for Gale-Shapley, ResOpt Constrained and ResOpt

eTable 4. Average percentage of positions filled and standard errors under Gale-Shapley vs ResOpt for ophthalmology and selected NRMP specialties

eFigure 1. The cumulative percentage of matches versus the program rank of the applicant under Gale-Shapley and ResOpt from the top matched to the sixth matched candidate if the program had six spots

eFigure 2. Percentage of total ophthalmology fellowship positions filled by Gale-Shapley and ResOpt from 2011 to 2020

eFigure 3. Percentage of ophthalmology sub-specialty fellowship positions filled by Gale-Shapley and ResOpt from 2011 to 2020

eFigure 4. The percentage of times partner A, B matched individually, in the same program and in the same city under Gale-Shapley (GS) versus ResOptCity for 1, 8, and 16 couples or 0.3%, 2.5%, and 5% of the applicant pool respectively

© 2025 Wu Y et al. *JAMA Network Open*.

eFigure 5. The difference between applicants' mean rank of their matched programs under Gale-Shapley and ResOpt

eAppendix 1. NRMP bootstrap

eTable 5. 2019 Reported total positions, matched positions, fill rate, versus fill rate and standard error for Gale-Shapley and ResOpt run on 10 bootstrapped rank lists

eAppendix 2. Comparison of specialty demographics

eTable 6. Applicant demographics for ophthalmology in 2019 and 2022 and available NRMP specialties in 2022

eTable 7. Applicant match rates for ophthalmology in 2019 and 2022 and available NRMP specialties in 2022

eAppendix 3. Comparison of educational background

eTable 8. Number of matched applicants and their percentage of total matched applicants by schooling

eTable 9. Number of applicants, percentage of all applicants, the number matches and the match rate for US graduating seniors versus all other sources for select specialties in 2019

eAppendix 4. Bootstrap of non-US senior applicants

eTable 10. Fill rate, average matched ranks for applicants and programs as well as the percentage of applicants matching their top 3 programs for Gale-Shapley and ResOpt for 10 runs

eAppendix 5. ResOpt run times

eFigure 6. Number of programs times number of applicants versus CPU run times of ResOpt with error bars

© 2025 Wu Y et al. *JAMA Network Open*.

eAppendix 6. Toy problem

eFigure 7. Rank lists for applicants A to F on the left and programs X to Z on the right

eReferences

This supplemental material has been provided by the authors to give readers additional information about their work.

© 2025 Wu Y et al. *JAMA Network Open*.

eTable 1. Demographics for ophthalmology and selected NRMP specialties. RadOnc = radiation oncology; ENT = otolaryngology; Derm = dermatology; Ophtho = ophthalmology; Ortho = orthopedic surgery; Surgery = general surgery; FamilyMed = family medicine; IM = internal medicine; NRMP = National Resident Matching Program

Specialty	#Programs	#Positions	#Applicants	#Matched	Median Interviews Matched	Median Interviews Unmatched	Avg Ranked
Plastics	78	172	234	172	15	8	10.6
RadOnc	92	207	316	177	14	3	7.5
ENT	120	328	462	328	13	7	10.7
Derm	141	477	966	472	9	5	5.1
Ophtho	116	485	648	484	12	4	8.9
Pathology	162	601	924	569	10	8	6.6
Ortho	175	755	1037	752	13	6	10
Radiology	210	1088	2460	1072	14	9	6.7
Surgery	304	1432	2563	1432	14	9	8.12
Pediatrics	220	2847	3638	2778	13	6	9.3
FamilyMed	641	4107	6652	3827	12	6	6.6
IM	564	8116	12527	7892	13	6	7.3
All NRMP	4780	32194	58229	30550	13	7	7.4

© 2025 Wu Y et al. *JAMA Network Open*.

eTable 2. Match results for ResOpt vs Gale-Shapley. ResOpt matches more applicants than Gale-Shapley. Moreover, ResOpt achieves lower mean ranks of matched counterparty for applicants and programs than Gale-Shapley.

	**Number of Applicants Matched**		**Applicant Mean Matched Rank**		**Program Mean Normalized Matched Rank**	
	Gale-Shapley	ResOpt	Gale-Shapley	ResOpt	Gale-Shapley	ResOpt
2011	457	461	2.75	2.32	2.85	2.56
2012	458	461	2.61	2.34	3.08	2.63
2013	455	460	2.66	2.35	3.04	2.67
2014	460	461	2.88	2.44	2.82	2.57
2015	464	465	2.98	2.43	2.82	2.55
2016	467	469	2.90	2.30	2.94	2.72
2017	462	468	2.87	2.55	3.36	2.85
2018	474	475	2.99	2.51	3.09	2.67
2019	484	485	2.76	2.38	3.10	2.67
2020	495	496	2.90	2.39	2.93	2.60
2021	498	499	3.05	2.39	2.68	2.63
**Overall**	5174	5200	2.85	2.40	2.97	2.65

© 2025 Wu Y et al. *JAMA Network Open*.

eTable 3. Cumulative percentage of applicants matching their top three ranked programs by year for Gale-Shapley, ResOpt Constrained and ResOpt. The difference in cumulative percentage between ResOpt and Gale-Shapley is positive every year and 7.1% overall.

Year	Matched rank	Gale-Shapley	ResOpt Constrained	ResOpt	ResOpt - Gale-Shapley
2011	3	73.7%	78.3%	80.9%	7.2%
2012	3	73.4%	76.4%	79.4%	6.0%
2013	3	75.2%	76.3%	78.9%	3.7%
2014	3	70.4%	75.2%	79.2%	8.7%
2015	3	70.0%	73.9%	77.6%	7.6%
2016	3	70.9%	75.8%	80.8%	9.9%
2017	3	69.3%	75.3%	75.6%	6.4%
2018	3	67.3%	71.7%	75.8%	8.5%
2019	3	73.8%	76.2%	79.8%	6.0%
2020	3	69.7%	73.8%	77.0%	7.3%
2021	3	66.7%	72.7%	77.4%	10.7%
**Overall**	3	70.9%	74.7%	78.4%	7.5%

© 2025 Wu Y et al. *JAMA Network Open*.

eTable 4. Average percentage of positions filled and standard errors under Gale-Shapley vs ResOpt for ophthalmology and selected NRMP specialties.

	Actual 2019						Gale-Shapley		ResOpt	
Specialty	Positions	Filled	% Filled	Positions	Gale-Shapley	ResOpt	% Filled	Std Err	% Filled	Std Err
Plastics	172	172	100%	170	168	169.8	98.94%	1.3%	100.00%	0.0%
RadOnc	207	177	86%	205	195.9	202.9	95.51%	0.6%	98.93%	0.3%
ENT	328	328	100%	325	323.4	325.4	99.39%	0.4%	100.00%	0.0%
Derm	477	472	99%	468	467.1	467.8	99.85%	0.2%	100.00%	0.0%
Ophtho	485	484	100%	485	481.9	485	99.36%	0.5%	100.00%	0.0%
Ortho	755	752	100%	735	732.8	735.1	99.69%	0.3%	100.00%	0.0%
Pathology	601	569	95%	610	595.2	606.3	97.65%	0.3%	99.47%	0.1%
Radiology	1088	1072	99%	1094	1093.2	1093.9	99.94%	0.1%	100.00%	0.0%
Surgery	1432	1432	100%	1432	1431.1	1431.8	99.95%	0.1%	100.00%	0.0%
Pediatrics	2847	2778	98%	2822	2798.4	2822.4	99.15%	0.2%	100.00%	0.0%
FamilyMed	4107	3827	93%	4102	4023.5	4061.6	98.09%	0.1%	99.02%	0.1%
IM	8116	7892	97%	8120	8012.5	8090.1	98.67%	0.1%	99.63%	0.1%
All NRMP	32194	30550	95%	32191	31543	31995	97.99%	0.1%	99.39%	0.1%

© 2025 Wu Y et al. *JAMA Network Open*.

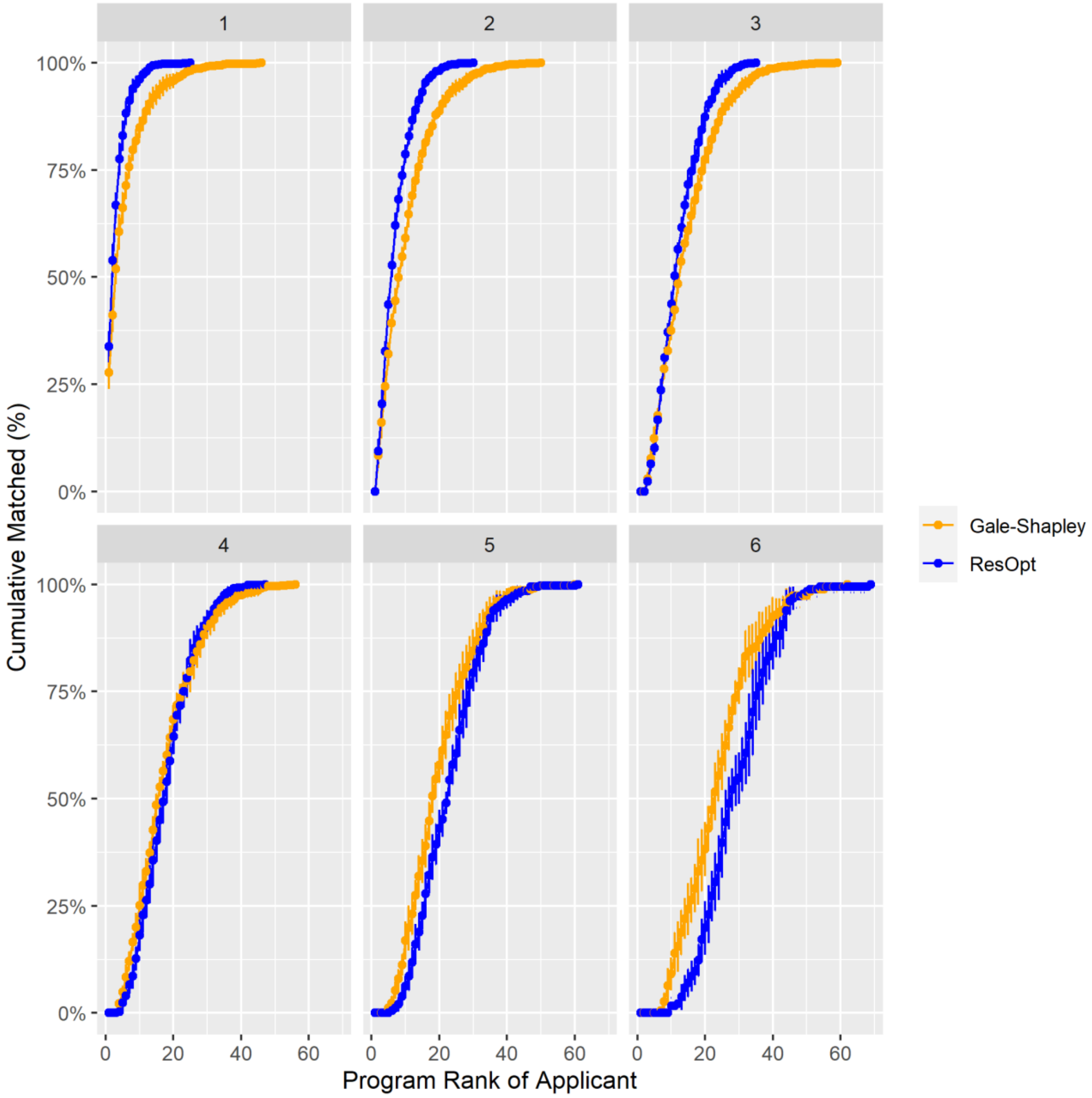

eFigure 1. The cumulative percentage of matches versus the program rank of the applicant under Gale-Shapley and ResOpt from the top matched to the sixth matched candidate if the program had six spots. The programs match statistically significantly more of their lower ranked or more desirable applicants in the first three spots under ResOpt than under Gale-Shapley.

© 2025 Wu Y et al. *JAMA Network Open*.

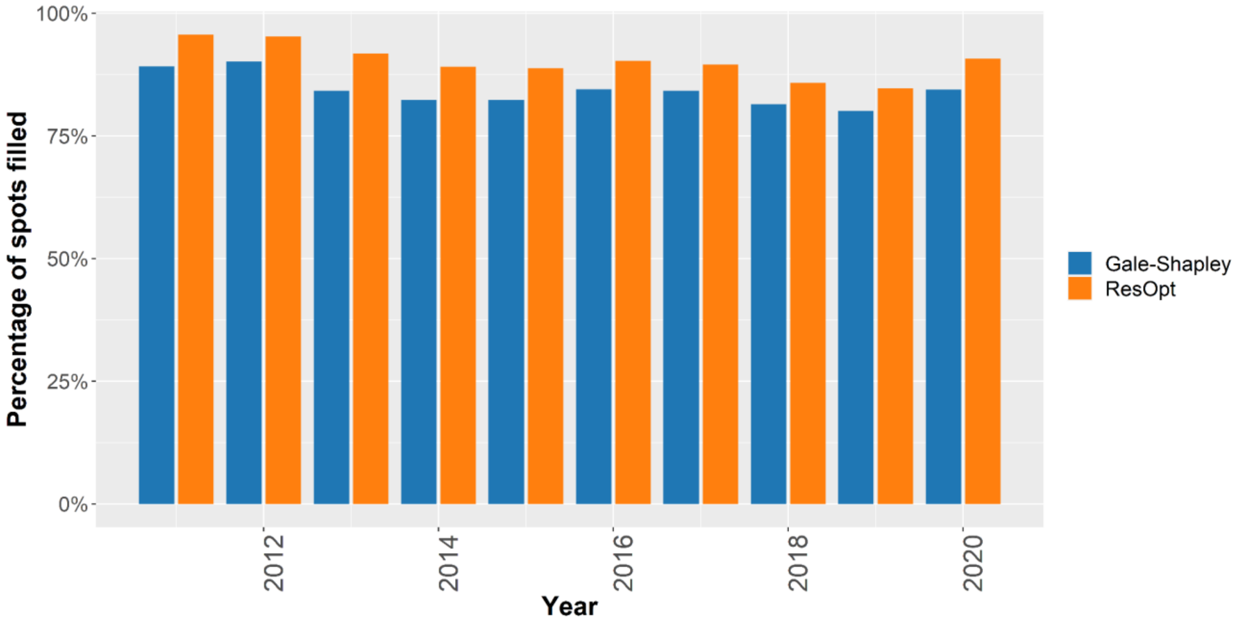

eFigure 2. Percentage of total ophthalmology fellowship positions filled by Gale-Shapley and ResOpt from 2011 to 2020. ResOpt consistently fills 5-10% more positions than Gale-Shapley

© 2025 Wu Y et al. *JAMA Network Open*.

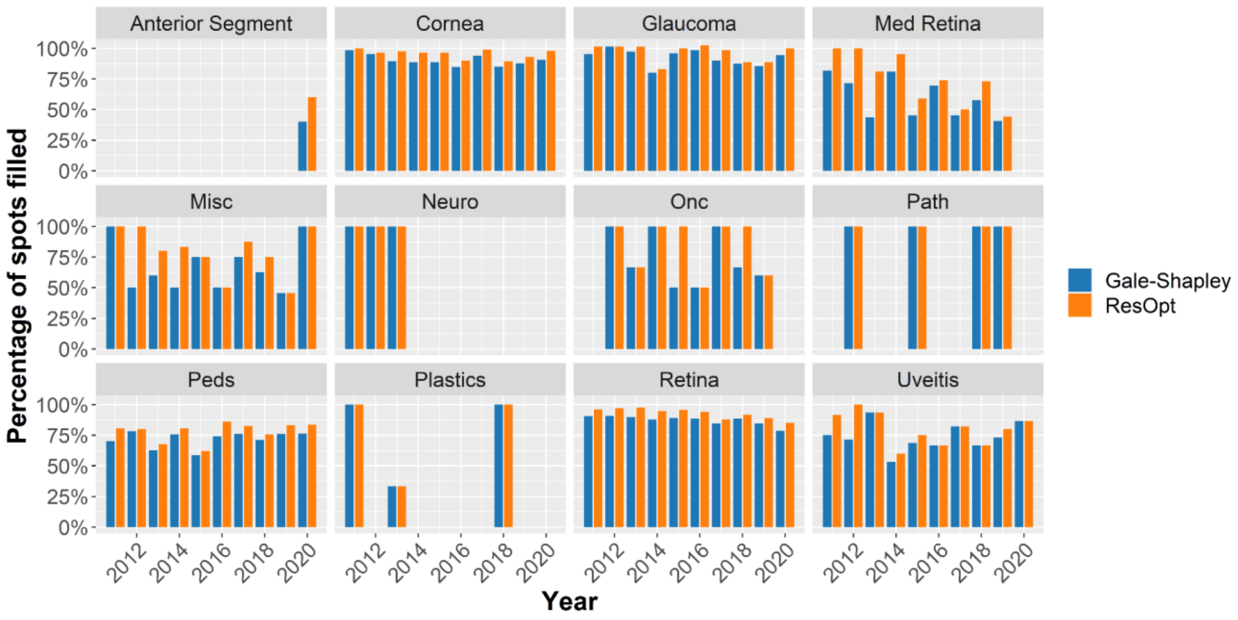

eFigure 3. Percentage of ophthalmology sub-specialty fellowship positions filled by Gale-Shapley and ResOpt from 2011 to 2020. ResOpt consistently fills more positions than Gale-Shapley.

© 2025 Wu Y et al. *JAMA Network Open*.

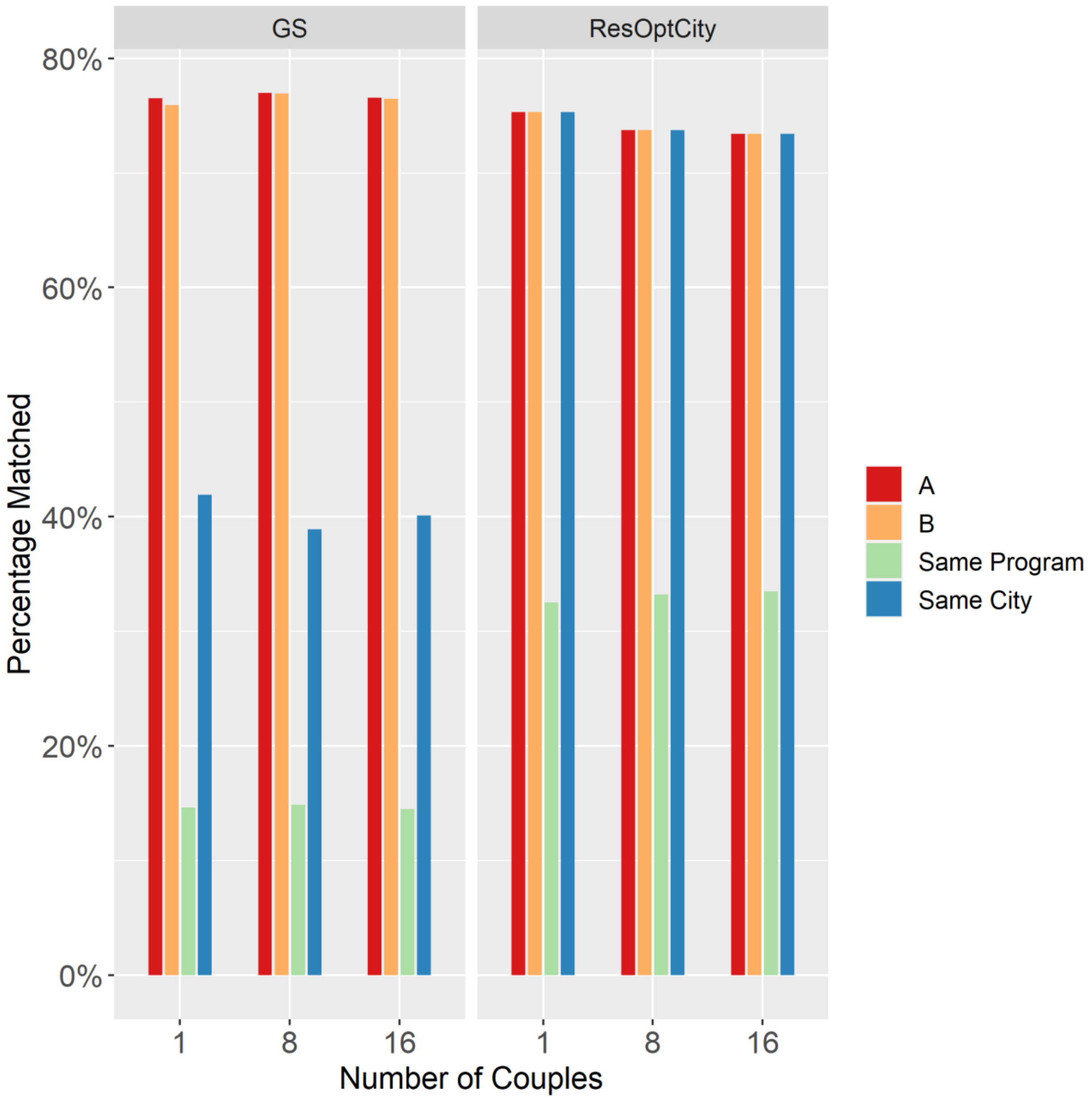

eFigure 4. The percentage of times partner A, B matched individually, in the same program and in the same city under Gale-Shapley (GS) versus ResOptCity for 1, 8, and 16 couples or 0.3%, 2.5%, and 5% of the applicant pool respectively.

© 2025 Wu Y et al. *JAMA Network Open*.

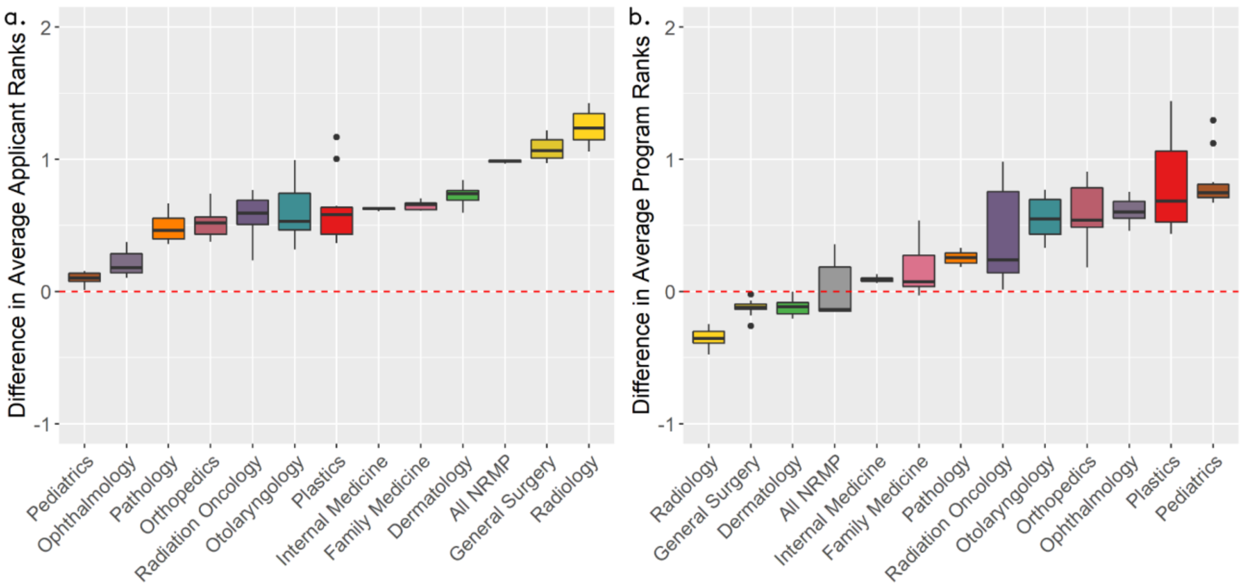

eFigure 5. The difference between applicants' mean rank of their matched programs under Gale-Shapley and ResOpt. **a)** The difference between applicants' mean rank of their matched programs under Gale-Shapley and ResOpt for selected NRMP specialties. A positive difference means ResOpt improved applicant outcome by lowering applicant mean ranks. The boxplot is over the 10 runs for each specialty and the boxplot whiskers represent the 95% confidence intervals of the difference in mean ranks. **b)** The difference between programs' mean rank of their matched applicants under Gale-Shapley and ResOpt. A positive difference means ResOpt improved program outcomes. The boxplot is over the 10 runs for each specialty and the boxplot whiskers represent the 95% confidence intervals of the difference in mean ranks.

© 2025 Wu Y et al. *JAMA Network Open*.

eAppendix 1. NRMP bootstrap

The NRMP specialty rank lists were bootstrapped from the 2019 Ophthalmology rank lists. The intuition is to rescale the ophthalmology rank lists to NRMP specialties in terms of the number of programs and applicants, as well as average number of ranks of programs ranked by applicants, while retaining characteristics of real rank list, such as:

1. Distribution of program ranks with a continuum of program popularities, with more popular programs being ranked by more applicants.
2. Furthermore, order of program rankings should be conserved when similarly popular. For example, let program *i* be a top ranked program and program *j* be a large program. Then program *i* might receive a lot of applicants because of its reputation, while program *j* will receive a lot of applicants because of its size. If *i* is more desirable than *j*, it will be relatively more desirable and receive higher rankings among the applicants that ranked it. The relative desirability of programs was determined using the PageRank algorithm(Page et al. 1999). In this case, the PageRank algorithm considers a rank as a referral link. Then programs will have high page rank when they are ranked by a lot of applicants, and these ranking applicants are ranked by a lot of programs and highly ranked.
3. Distribution of applicant ranks with a continuum of applicant popularities, with more popular applicants being ranked by more programs.
4. Similarly, the relative desirability of applicants was determined by PageRank. We note that the relative desirability of applicants, unlike that of programs, was fairly compact and did not vary widely.
5. Distribution of sparsity of program and applicant ranks.

Specifically, the bootstrap was implemented as follows:

1. The 2019 Ophthalmology program and applicants rank lists were turned into 2 rank matrices, each of dimension $p*n$, where p is the number of programs and n the number of applicants. One rank matrix, R_p, is for program ranks of applicants, and the other, R_a, applicant ranks of programs. Element x_{ij} of R_p is the integer rank by program *i* for applicant *j*, or NAN if program *i* did not rank applicant *j*. Similarly, x_{ij} of R_a is the integer rank by applicant *j* for program *i*, or NAN if applicant *j* did not rank program *i.*
2. Then for a NRMP specialty *k*, with p_k programs and n_k applicants, program and applicant rank matrices will be matrices of dimension p_k*n_k.
3. R_{pk}, the program rank matrix, is first initialized by sampling p_k rows from R_p with replacement. This results an intermediate matrix R_p' of dimension, p_k*n. Then, n_k columns are sampled from R_p', resulting in a R_{pk} with dimension p_k*n_k. Sampling R_{pk} in this way retains the distribution of program ranks in terms of sparsity.
4. Similarly, R_{ak}, the applicant rank matrix, is created by sampling p_k rows from R_a with replacement to get an intermediate matrix R_a', and then sampling n_k columns from R_a'

© 2025 Wu Y et al. *JAMA Network Open*.

resulting in R_{ak} with dimension p_k*n_k. This preserves the sparsity structure of applicant ranks,

5. Next, the vector of number spots, s, for each program from ophthalmology were first expanded by using the same sample indices from step 3. Then the number of spots were adjusted based on r, the ratio of the total number of spots in ophthalmology to the total number of spots in specialty k times p/p_k. The new vector of program spots, s_k, is rounded to the nearest integer number of spots, with the caveat that no program has 0 spots.
6. Then, the ranks in the rank matrices, R_{pk} and R_{ak}, are updated to retain program and applicant desirability characteristics and avoid ties in ranks.
7. The applicant ranks corresponding to columns of R_{ak}, i.e. the p_k program ranks by individual applicants, are updated in 3 steps.
8. In the first step, for each applicant j the number of programs they rank, t_j is calculated to be the initial t_{j_init}, the column-wise sum of R_{ak}, multiplied by the ratio of the NRMP reported average number of programs ranked in specialty k to the average number of programs ranked in ophthalmology times n_k/n. This ensures that the average number of ranks in the updated applicant rank matrix R_{ak} is consistent with the NRMP reported average number of ranked programs.
9. In the second step, for each applicant, t_j programs to be ranked are sampled according to program popularity, specifically the number of times the programs were ranked (row-wise sum of R_{ak}), which is then normalized so that more popular programs are proportionately more likely to be sampled.
10. In the third step, the rank order of the sampled programs are determined by sampling according to the vector of program desirability by calculated using the PageRank algorithm on R_{pk} and R_{ak}. More desirable programs are more likely to be sampled first and receive more desirable or lower ordinal ranks.
11. Steps 8-10, are repeated for each of the n_k applicants resulting in an updated R_{ak}. These sampling steps retain distribution characteristics of program popularity and relative desirability.
12. Next, The program ranks corresponding to rows of R_{pk}, i.e. the n_k applicant ranks, are updated in 2 steps.
13. In the first step, each program will rank 95% of the applicants that ranked them in the updated R_{ak} from step 10. This mimics the attrition between program and applicant interviews, as we found applicants nearly always rank all programs where they interviewed, but programs do not always rank all applicants they interviewed.
14. In the second step, the rank order of the applicants are sampled according to the vector of applicant desirability by the PageRank algorithm on R_{pk} and R_{ak}.
15. Steps 13 and 14, are repeated for each of the p_k programs to yield an updated R_{pk}. These sampling steps retain distribution characteristics of applicant popularity and relative desirability.

© 2025 Wu Y et al. *JAMA Network Open*.

These bootstrap steps allowed the construction of rank matrices for individual NRMP specialties. The rank matrices were checked after construction to check that matched reported NRMP summary statistics for the specialty in 2019 in terms of:

1. Number of applicants, n_k
2. Number of programs, p_k
3. Total number of spots, sum(s_k)
4. The average number of programs ranked by applicants

Finally, rank matrices for the NRMP match as a whole are achieved by stacking the specialty rank matrices block diagonally.

To assess whether these specialty rank matrices captured realistic dynamics of the match, we bootstrapped each specialty 10 times and ran the Gale-Shapley and ResOpt algorithms on the bootstrapped rank matrices and compared match statistics in terms of positions filled.

	Actual 2019			Gale-Shapley		ResOpt	
Specialty	Positions	Filled	% Filled	% Filled	Std Err	% Filled	Std Err
Plastics	172	172	100%	98.94%	1.3%	100.00%	0.0%
ENT	328	328	100%	99.39%	0.4%	100.00%	0.0%
Derm	477	472	99%	99.85%	0.2%	100.00%	0.0%
Ophtho	485	484	100%	99.36%	0.5%	100.00%	0.0%
Ortho	755	752	100%	99.69%	0.3%	100.00%	0.0%
Pathology	601	569	95%	97.65%	0.3%	99.47%	0.1%
Radiology	1088	1072	99%	99.94%	0.1%	100.00%	0.0%
Surgery	1432	1432	100%	99.95%	0.1%	100.00%	0.0%
Pediatrics	2847	2778	98%	99.15%	0.2%	100.00%	0.0%
FamilyMed	4107	3827	93%	98.09%	0.1%	99.02%	0.1%
IM	8116	7892	97%	98.67%	0.1%	99.63%	0.1%
All NRMP	32194	30550	95%	97.99%	0.1%	99.39%	0.1%

eTable 5. 2019 Reported total positions, matched positions, fill rate, versus fill rate and standard error for Gale-Shapley and ResOpt run on 10 bootstrapped rank lists.

Overall, for most NRMP specialties, our Gale-Shapley fill rates plus/minus 2*standard errors are within the actual reported NRMP fill rate, which also uses Gale-Shapley. The two outlier specialties were pathology and family medicine. In these cases, our bootstrapped rank lists may be too optimistic and resulted in higher than reported match rates. However for fill rates across

© 2025 Wu Y et al. *JAMA Network Open*.

all specialties, ResOpt always did better, as it is more robust to sparse and short rank lists as shown in Figure 3.

We also checked the percentage of applicants that matched their top 3 ranked programs in the bootstrapped NRMP experiments. For matched applicants, on average 72.6% matched their top 3 programs. This is similar to the reported 76.9% US Seniors across all NRMP specialties that matched their top 3 in 2019, according to Figure 7 of the Results and Data Report of the 2019 Main Residency Match(National Resident Matching Program 2019). The bootstrapped 72.9% is slightly lower than 76.9% as all the bootstrapped specialties, except Family Medicine, are very competitive specialties with higher than reported average NRMP fill rates and might not be representative of NRMP overall. Unfortunately, the NRMP does not report top 3 program match percentages at the specialty level to allow a more detailed comparison.

© 2025 Wu Y et al. *JAMA Network Open*.

eAppendix 2. Comparison of specialty demographics

NRMP only reported demographics for applicants and their match rates in select specialties for 2022, (Laurie S. Curtin, Risë B. Goldstein, ...). We compared these to 2022 Ophthalmology(Ophthalmology Residency February 2023), as well as to 2019 Ophthalmology, since 2019 Ophthalmology rank lists were used to bootstrap to NRMP. Applicant demographics in 2019 and 2022 Ophthalmology were similar. One minor difference was that Hispanic was considered separately from race in Ophthalmology in 2022 so that the row sums to more than 100%. However, this separate definition of Hispanic is consistent with NRMP usage. eTable 6 shows the demographic breakdown for applicants to Ophthalmology and available NRMP specialties. Ophthalmology had comparable applicant demographics to Radiology, Pediatrics and Family Medicine.

eTable 6. Applicant demographics for ophthalmology in 2019 and 2022 and available NRMP specialties in 2022. Note Hispanic is defined separately from race.

Specialty	White	Asian	Black	Other	Declined	Hispanic
Ophtho 2019	51.9%	30.8%	3.6%	2.2%	3.4%	8.2%
Ophtho 2022	51.0%	29.0%	6.0%	5.0%	9.0%	9.0%
Ortho	68.7%	16.8%	7.1%	2.8%	3.2%	9.2%
Radiology	53.4%	29.6%	5.7%	5.1%	5.5%	9.7%
Surgery	57.5%	23.7%	7.9%	4.4%	4.7%	13.5%
Pediatrics	56.9%	26.0%	8.3%	4.5%	3.0%	11.8%
FamilyMed	49.9%	28.5%	10.0%	4.7%	4.7%	11.7%
IM	37.7%	41.2%	8.4%	7.4%	4.4%	10.4%

Next, applicant outcomes in terms of match rates were compared in eTable 7. For Ophthalmology, SF Match only reported matched applicants as a percentage of total match by demographics. However, Ophthalmology match rates were easily derived: match rate for ethnicity = (ethnicity as % of total matched * total number matched)/(ethnicity as % of total

© 2025 Wu Y et al. *JAMA Network Open*.

applicants * total applicant number). All specialties had higher match rates for white applicants. Ophthalmology had similar match rates for White matched applicants, the largest contingent of applicants, to Radiology and Surgery. Moreover, Ophthalmology had similar match rates for non-White applicants to Radiology, Surgery, Family Medicine and Internal Medicine.

eTable 7. Applicant match rates for ophthalmology in 2019 and 2022 and available NRMP specialties in 2022. Note Hispanic is defined separately from race.

Specialty	White	Asian	Black	Other	Declined	Hispanic
Ophtho 2019	80.2%	70.1%	63.4%	93.4%	76.1%	72.4%
Ophtho 2022	76.1%	73.4%	88.7%	76.1%	67.6%	67.6%
Ortho	66.8%	53.4%	65.5%	58.8%	46.2%	57.5%
Radiology	79.9%	78.2%	70.4%	65.1%	67.7%	76.7%
Surgery	73.5%	66.3%	64.2%	56.0%	58.2%	57.9%
Pediatrics	94.8%	85.3%	81.4%	78.1%	81.0%	86.3%
FamilyMed	88.9%	72.4%	66.5%	66.7%	65.3%	78.4%
IM	86.9%	73.8%	63.7%	66.7%	72.2%	77.9%

© 2025 Wu Y et al. *JAMA Network Open*.

eAppendix 3. Comparison of educational background

We compared the educational background of matched applicants for Ophthalmology and select NRMP specialties in 2019, in eTable 8. Some NRMP specialties are reported as PGY1 and PGY2, following Table 2 of the 2019 NRMP Results and Data Report of the Main Residency Match(National Resident Matching Program 2019). Ophthalmology is similar to many specialties with close to 90% of matched applicants being US graduating seniors. Furthermore, it is like Plastics, RadOnc, ENT, and Ortho in having less than 5% matched applicants be former US graduates or Osteopathic graduates.

© 2025 Wu Y et al. *JAMA Network Open*.

eTable 8. Number of matched applicants and their percentage of total matched applicants by schooling.

	Total Positions	Total Matched	US Seniors		US Grads		Osteo		International	
**Speciality**			**Matched**	**% Match**	**Matched**	**% Match**	**Matched**	**% Match**	**Matched**	**% Match**
Ophtho	485	484	433	89.50%	16	3.30%	18	3.70%	17	3.50%
Plastics	172	172	158	91.90%	8	4.70%	2	1.20%	4	2.30%
RadOnc PGY1	15	14	13	92.90%	0	0.00%	0	0.00%	1	7.10%
RadOnc PGY2	192	163	147	90.20%	5	3.10%	5	3.10%	6	3.70%
ENT	328	328	308	93.90%	0	0.00%	13	4.00%	7	2.10%
Derm PGY1	30	28	23	82.10%	0	0.00%	3	10.70%	2	7.10%
Derm PGY2	447	444	370	83.30%	35	7.90%	30	6.80%	9	2.00%
Ortho	755	752	693	92.20%	34	4.50%	15	2.00%	10	1.30%
Pathology	601	569	201	35.30%	29	5.10%	57	10.00%	282	49.60%
Radiology PGY1	123	122	73	59.80%	2	1.60%	25	20.50%	22	18.00%
Radiology PGY2	965	950	665	70.00%	44	4.60%	132	13.90%	109	11.50%
Surgery	1432	1432	1053	73.50%	83	5.80%	143	10.00%	153	10.70%
Pediatrics	2847	2778	1715	61.70%	34	1.20%	503	18.10%	526	18.90%
FamilyMed	4107	3827	1601	41.80%	126	3.30%	986	25.80%	1114	29.10%
IM	8116	7892	3366	42.70%	119	1.50%	1202	15.20%	3205	40.60%

To better understand the impact of schooling on applicant outcomes, we analysed the match rate of US Seniors versus other applicants in eTable 9; NRMP only breaks down applicant numbers by US Seniors versus others and does not provide fine-grained detail as eTable 8. From eTable 9, Ophthalmology had similar percentages of non-US Senior applicants as Plastics and Ortho. For all specialties, US graduating seniors had much higher match rates. Ophthalmology had similar US Senior match rates to Plastics, ENT, Derm, Ortho and Surgery. Moreover, Ophthalmology had comparable or lower match rates for non-US Seniors to most

© 2025 Wu Y et al. *JAMA Network Open*.

specialties. It only had substantially higher match rates than the PGY1 specialties, which almost exclusively matched US Seniors. Therefore, there is no evidence that Ophthalmology is more exclusive of non-US Seniors applicants compared to NRMP.

eTable 9. Number of applicants, percentage of all applicants, the number matches and the match rate for US graduating seniors versus all other sources for select specialties in 2019.

	Total Applicants	US Seniors				Other			
Speciality		Applicants	% Applicants	Matched	Match Rate	Applicants	% Applicants	Matched	Match Rate
Ophtho	649	512	78.90%	433	84.60%	137	21.10%	51	37.20%
Plastics	234	188	80.30%	158	84.00%	46	19.70%	14	30.40%
RadOnc PGY1	124	115	92.70%	13	11.30%	9	7.30%	1	11.10%
RadOnc PGY2	192	163	84.90%	147	90.20%	29	15.10%	16	55.20%
ENT	462	398	86.10%	308	77.40%	64	13.90%	20	31.30%
Derm PGY1	265	217	81.90%	23	10.60%	48	18.10%	5	10.40%
Derm PGY2	701	488	69.60%	370	75.80%	213	30.40%	74	34.70%
Ortho	1,037	830	80.00%	693	83.50%	207	20.00%	59	28.50%
Pathology	924	216	23.40%	201	93.10%	708	76.60%	368	52.00%
Radiology PGY1	940	595	63.30%	73	12.30%	345	36.70%	49	14.20%
Radiology PGY2	1,520	925	60.90%	665	71.90%	595	39.10%	285	47.90%
Surgery	2,563	1,388	54.20%	1,053	75.90%	1,175	45.80%	379	32.30%
Pediatrics	3,638	1,901	52.30%	1,715	90.20%	1,737	47.70%	1,063	61.20%
FamilyMed	6,652	1,927	29.00%	1,601	83.10%	4,725	71.00%	2,226	47.10%
IM	12,527	3,966	31.70%	3,366	84.90%	8,561	68.30%	4,526	52.90%

© 2025 Wu Y et al. *JAMA Network Open*.

eAppendix 4. Bootstrap of non-US senior applicants

To make sure ResOpt is not susceptible to non-US Senior applicants who tend to have lower number of interviews and thereby lower number of programs ranking them, we ran an experiment by bootstrapping from the 2019 Ophthalmology rank lists to have characteristics of non-US Senior applicants. This was done by first randomly selecting 60% of applicants, to match the non-US Senior applicant rates in Internal Medicine. Next, for these 60% applicants, 30% of the programs that previously ranked them were randomly selected to no longer rank them. This drop off in ranks matches the difference in median number of ranks for matched US Senior (13) to non-US Senior (9) in NRMP in 2019 (Results of the 2019 NRMP Applicant Su...). We bootstrapped the rank lists in this way 10 times, and for each bootstrap, Gale-Shapley and ResOpt were run on the adjusted rank lists. The resulting match statistics are shown in eTable 10. There are three main takeaways from these 10 experiments. First, ResOpt filled more positions than Gale-Shapley in each run. Second, ResOpt achieved lower average matched ranks for applicants and programs in every run. Finally, ResOpt matched more applicants to their top 3 choice programs than Gale-Shapley for all 10 runs. These results show that ResOpt will be more robust to scenarios where a certain group of applicants will be less ranked than others. This group can be non-US Seniors, but could also easily apply to under-represented minorities, who might also receive less interview and ranking opportunities.

© 2025 Wu Y et al. *JAMA Network Open*.

eTable 10. Fill rate, average matched ranks for applicants and programs as well as the percentage of applicants matching their top 3 programs for Gale-Shapley and ResOpt for 10 runs.

		Gale-Shapley					ResOpt				
				Average Ranks					Average Ranks		
Run	Positions	Matched	Fill Rate	Applicants	Programs	Top 3 %	Matched	Fill Rate	Applicants	Programs	Top 3 %
1	485	481	99.20%	2.9	3.3	71.10%	485	100.00%	2.6	3.0	75.50%
2	485	480	99.00%	3.1	3.3	66.80%	485	100.00%	2.7	3.0	74.80%
3	485	483	99.60%	3.0	3.4	69.30%	485	100.00%	2.6	3.0	75.30%
4	485	484	99.80%	2.9	3.3	71.10%	485	100.00%	2.6	3.0	76.50%
5	485	480	99.00%	2.9	3.3	69.10%	485	100.00%	2.6	3.0	75.90%
6	485	480	99.00%	2.9	3.3	69.50%	485	100.00%	2.8	2.8	72.40%
7	485	480	99.00%	3.0	3.3	70.30%	485	100.00%	2.6	3.0	75.70%
8	485	482	99.40%	2.9	3.4	71.30%	485	100.00%	2.6	3.0	74.60%
9	485	480	99.00%	2.9	3.3	68.70%	485	100.00%	2.6	3.0	74.60%
10	485	481	99.20%	2.9	3.3	72.40%	485	100.00%	2.6	2.9	76.30%

© 2025 Wu Y et al. *JAMA Network Open*.

eAppendix 5. ResOpt run times

We bootstrapped rank lists of different sizes to analyze the CPU run times. The base rank list setting was:

1. 100 Programs
2. 450 Total Positions, with 5% 1-spot, 10% 2-spot, 15% 3-spot, 20% 4-spot, 20% 5-spot, 15% 6-spot, 10% 7-spot and 5% 8-spot programs
3. Programs rank on average 10 applicants per spot
4. 600 Applicants
5. Each applicant ranks every program that ranked them, which replicated current match characteristics.

This base setting was scaled down to 50 Programs, 300 Applicants and 225 positions, and up to 5000 Programs, 30000 Applicants and 22500 positions, which is similar in size to NRMP. For each program and applicant setting, 10 rank lists were bootstrapped, and ResOpt was run on each rank list using one core of an AMD EPYC 7302 16-Core Processor with 16GB RAM. ResOpt results were verified to have matched the correct number of positions and CPU run times recorded. eFigure 6 shows the CPU times versus the number of program times the number of applicants. Even for matches the size of NRMP, ResOpt ran in less than 6 seconds.

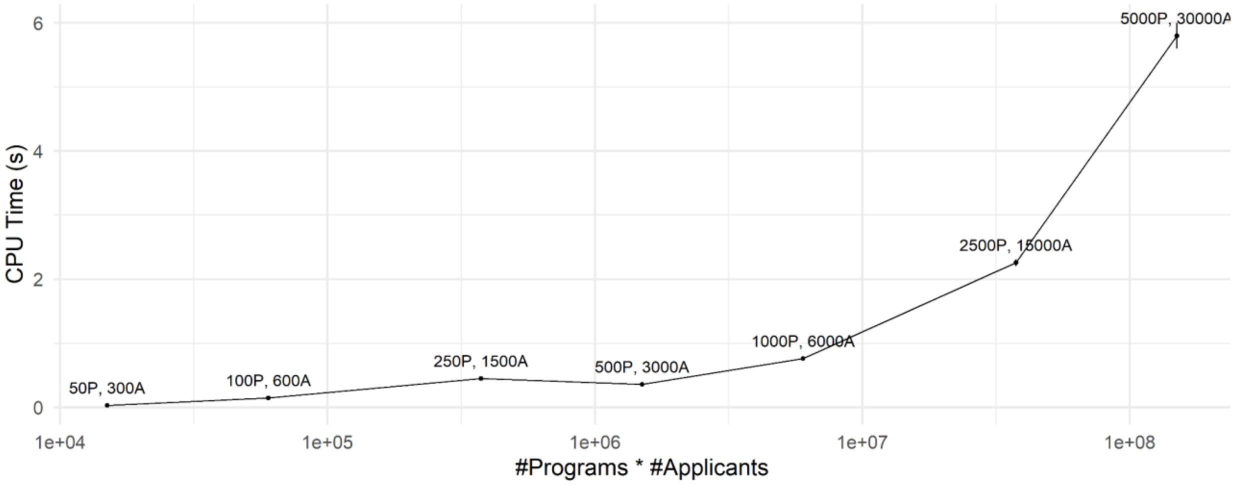

eFigure 6 Number of programs times number of applicants versus CPU run times of ResOpt with error bars. Error bars for most settings are small.

© 2025 Wu Y et al. *JAMA Network Open*.

eAppendix 6. Toy problem

We considered toy problems with generated rank lists for 3 programs and 6 applicants. Let the programs be denoted X, Y, and Z, and the 6 applicants be denoted A to F. Also let X and Y each have 1 spot and Z has 2 spots. eFigure 6 shows an example with generated rank lists. The top row is the "stable-marriage" from Gale-Shapley for this example, and the bottom row is the ResOpt match. ResOpt does 1 rank worse for applicant A and 2 better for applicant F, for a net 1 rank gain on the applicant side. For programs, ResOpt improves program X's match by 1 normalized rank and program Z's by half a normalized rank as Z has two applicants. Using normalized ranks avoids optimizing for larger programs at the expense of smaller programs. We exhaustively enumerated all possible match configurations for these rank lists and found 6 out of 156 valid configurations to achieve lower total matched ranks than Gale-Shapley. This experiment was repeated and approximately 5% of possible configurations for toy setups with 3 programs and 6 applicants are superior to Gale-Shapley in terms of total matched ranks. Note in these toy problems, all programs ranked all applicants and vice versa. In practice, applicants and programs can only rank a subset of their counterparties. However, exhaustive enumeration of matches with more programs and applicants soon become computationally intractable.

© 2025 Wu Y et al. *JAMA Network Open*.

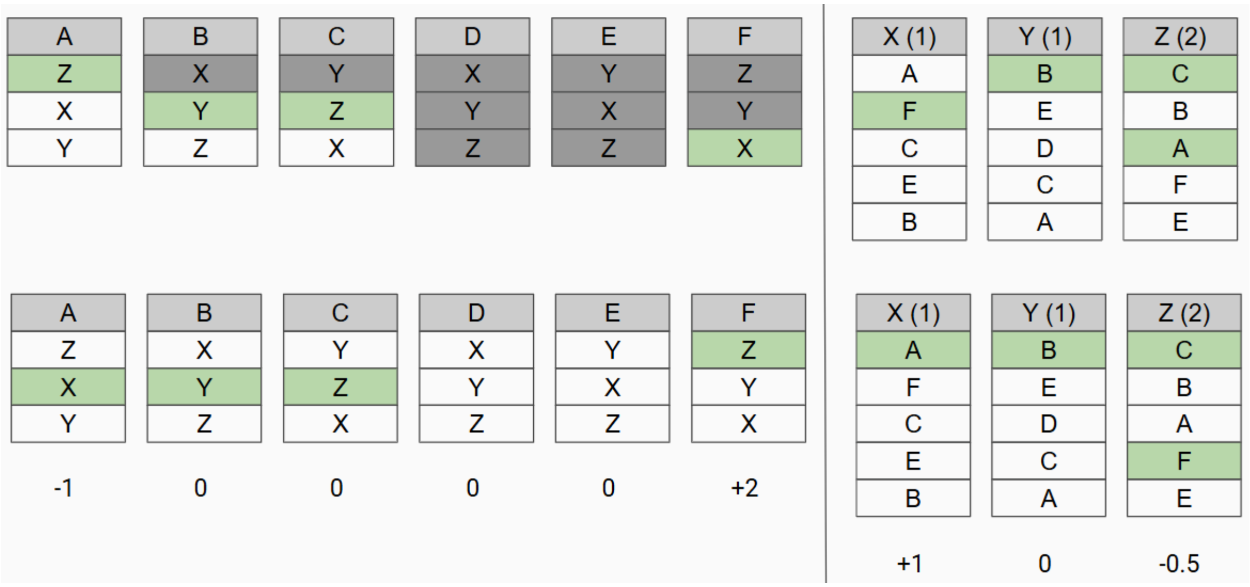

eFigure 7 Rank lists for applicants A to F on the left and programs X to Z on the right. The matches are shown in green with Gale-Shapley on the top row and ResOpt on the bottom. The dark grey colored boxes show applicants that tried to match programs, but were later rejected as other more preferred applicants were accepted under Gale-Shapley. The numbers at the bottom reflect net ranks gained and lost using ResOpt and Gale-Shapley.

© 2025 Wu Y et al. *JAMA Network Open*.

eReferences

1. Laurie S. Curtin, Risë B. Goldstein, Donna L. Lamb. n.d. “NRMP Applicant Demographics and the Transition to Residency.” https://www.nrmp.org/wp-content/uploads/2023/02/Demographic-data-perspectives-paper_FINAL.pdf#:~:text=Applicant,Hispanic%2FLatinx%2FSpanish.

2. National Resident Matching Program. 2019. Results and Data Main Match 2019. National Resident Matching Program.

3. “Ophthalmology Residency February 2023.” n.d. https://sfmatch.org/files/d8eff88d08bd4b58a228ed60c0b6dba7.

4. Page, Lawrence, Sergey Brin, Rajeev Motwani, and Terry Winograd. 1999. “The PageRank Citation Ranking: Bringing Order to the Web.” Stanford InfoLab. http://ilpubs.stanford.edu:8090/422/.

5. “Results of the 2019 NRMP Applicant Survey.” 2019. NRMP. https://mk0nrmp3oyqui6wqfm.kinstacdn.com/wp-content/uploads/2019/06/Applicant-Survey-Report-2019.pdf.

© 2025 Wu Y et al. *JAMA Network Open*.
