# Supplementary material for: Improving Residency Matching Through Computational Optimization
Source: JAMA Netw Open. 2025 Jun 23;8(6):e2517077. doi: 10.1001/jamanetworkopen.2025.17077 (PMC12186513; doi:10.1001/jamanetworkopen.2025.17077)
Supplement: Supplement 2. — Data Sharing Statement [file jamanetwopen-e2517077-s002.pdf]

Data Sharing Statement

Wu. Improving Residency Matching Through Computational Optimization. *JAMA Netw Open*. Published June 23, 2025. doi:10.1001/jamanetworkopen.2025.17077

Data
Data available: No

Additional Information
Explanation for why data not available: The de-identified match data used in this manuscript were provided under research agreement from the Association of University Professors of Ophthalmology. The authors do not have authority to share this dataset.
